# Restructuring surgical training after COVID-19 pandemic: A nationwide survey on the Italian scenario on behalf of the Italian polyspecialistic young surgeons society (SPIGC)

**DOI:** 10.3389/fsurg.2022.1115653

**Published:** 2023-01-11

**Authors:** Gaetano Gallo, Eleonora Guaitoli, Fabio Barra, Arcangelo Picciariello, Alessandro Pasculli, Alessandro Coppola, Davide Pertile, Roberto Luca Meniconi, SPIGC Surgical Training Working Group, Federico Berton

**Affiliations:** ^1^ Department of Surgical Sciences, La Sapienza” University of Rome, Rome, Italy; ^2^ Department of Surgery, A. Perrino Hospital, Brindisi, Italy; ^3^ Academic Unit of Obstetrics and Gynecology, IRCCS Ospedale Policlinico San Martino, Genova, Italy; ^4^ Department of Emergency and Organ Transplantation, University Aldo Moro, Bari, Italy; ^5^ Department of Biomedical Sciences and Human Oncology - Unit of Endocrine, Digestive and Emergency Surgery, University “A. Moro” of Bari, Policlinic of Bari, Bari, Italy; ^6^ Department of Surgery, La Sapienza” University of Rome, Rome, Italy; ^7^ Department of Surgery, Policlinico San Martino, Genova, Italy; ^8^ Department of General Surgery and Liver Transplantation, San Camillo Forlanini Hospital, Rome, Italy

**Keywords:** surgical training, COVID-19 pandemic, trainee, training programme, survey

## Abstract

**Introduction:**

The COVID-19 pandemic has led to the disruption of surgical training. Lack of communication, guidelines for managing clinical activity as well as concerns for safety in the workplace appeared to be relevant issues. This study aims to investigate how surgical training has been reorganized in Italy, almost 2 years after the outbreak of COVID-19 pandemic.

**Materials and methods:**

A 16-item-electronic anonymous questionnaire was designed through SurveyMonkey^©^ web application. This survey was composed of different sections concerning demographic characteristics and impacts of the second COVID-19 pandemic wave on surgical and research/didactic activities. Changes applied in the training programme and activities carried out were also investigated. The survey was carried out in the period between June and October 2021.

**Results:**

Four hundred and thirty responses were collected, and 399 were considered eligible to be included in the study analysis. Three hundred and thirty-five respondents continued working in Surgical Units, with a significant reduction (less than one surgical session per week) of surgical sessions in 49.6% of them. With concern to didactic and research activities, 140 residents maintained their usual activity, while 116 reported a reduction. A sub-group analysis on resident moved to COVID-19 departments showed a reduction of research activities in 35% of them. During the period considered in this survey, the surgical training program was not substantially modified for most of participants (74.6%).

**Conclusion:**

Our survey demonstrated that surgical residency programs haven't improved 2 years after the beginning of the pandemic. Further improvements are needed to guarantee completeness of surgical training, even in emergency conditions.

## Introduction

In January 2022, COVID-19 globally reached almost 350 million cases, accounting for around 5.5 million deaths (1). Following UK, France, Russia and Turkey, Italy was the fifth European state for total COVID-19 cases, recording more than ten million people that tested positive and 144,000 deaths (2).

The first COVID-19 outbreak lead Health Systems to face a burden never seen before. The drastic changes in human resources negatively impacted healthcare workers everyday life. Surgical units suffered an unavoidable impact, with discontinuation of elective non-oncological procedures, outpatient clinics, and endoscopy services (3–6). Considering the above, also surgical education programs were drastically affected by the pandemic (7, 8).

In 2020, an international survey-based study, including 15 specialities and 34 countries, assessed the global impact of the COVID-19 pandemic on surgical training (9), demonstrating a severe impact on all of its aspects. Interestingly, trainees from Europe reported worse consequences than those from Asia and Australia.

In our previous survey (10), among 800 responses collected, almost 35% and 27% of respondents declared they were experiencing, respectively, complete interruption of surgical and clinical activities. A subgroup analysis, comparing the COVID-19 impact on clinical activities with demographics data demonstrated a statistically significant difference regarding the various surgical specialities and Italian regions.

Lack of communications and coordination from the institutions, and guidelines for managing clinical activities during the pandemic have been identified as significant negative factors for surgical training during COVID-19 pandemic (11, 12).

Overall, anxiety for rising difficulties in career progression and concerns for safety on the workplace related to COVID-19 pandemic were underlined as relevant issues potentially affecting residents’ training (13, 14).

The present study aims to investigate how surgical training was reorganised in Italy almost 2 years after the outbreak of COVID-19 Pandemic.

## Methods

This survey was carried out between June and October 2021 by the Italian Polyspecialistic Society of Young Surgeons (SPIGC). It consists of an anonymous questionnaire created through SurveyMonkey^©^ web application (SVMK Inc., One Curiosity Way, San Mateo, United States) (15). The aim of the survey was explained to all participants with a brief introduction. Participants were asked to sign a privacy policy consent. Survey participation was voluntary, and no incentives were offered. No institutional review board approval was required. This survey was registered in clinicaltrias.gov (NCT04338945).

The survey was composed of three sections. The first section included 5 Questions (Q) concerning whether or not participants belong to a surgical training programme, demographics, level of training and type of surgical activity routinely performed (Q1–Q7). The second section was concerned with the impact of second wave of the COVID-19 pandemic on clinical, surgical and research activities (Q8–Q10). The third section focused on the activities carried out during the second wave, the changes applied in the training programme and COVID-19 vaccine (Q11–Q16).

These questions were selected and collected by the authors, with the aim of providing an accurate scenario of COVID-19’s impact on trainees’ activities.

The survey was promoted through a mailing list, instant message services, and through the SPIGC official Facebook, Instagram, and LinkedIn accounts.

Italian surgical residents coming from any surgical specialty and attending all years of training program were considered eligible for the survey's analysis. The eligibility has no relation to the residents’ curricular activities. As previously done (10), the study sample aimed to reach approximately 5% of Italian residents in surgical specialties concerning the annual number of residency scholarship places from 2014 to 2019, and the annual drop-out percentage of surgical trainees (16). All participants were informed that the results of the survey would have been used for further statistical evaluation and scientific publication. Anonymity was guaranteed by study design.

Results of the survey were reported according to the CHERRIES Guidelines (17).

### Statistical analysis

All the answers collected and included in the study were processed, and results were summarized as numbers (*n*) and percentages (%), separately for each question. A *p*-value < 0.05 was considered statistically significant. All the analyses were performed with RStudio (Version 1.1.463-© 2009–2018 RStudio, Inc.).

## Results

### Study population

Out of 430 participants, 399 (92.7%) were included in the study. The response rate for specific questions ranged from 92.2% to 100%.

Overall, trainees answering to this survey were in 49.1% of cases male (*n* = 196) and in 50.9% female (*n* = 203). Most residents (56.4%) were attending a training program in general surgery, followed by plastic surgery (7.77%). The complete subdivision according to surgical specialties has been reported in Table 1. One hundred ninety-four (49.2%) responders were 28 (27–30) years old, 136 (34.5%) were 26 (25–27) years old, and 63 (16%) were aged over 30 years.

**Table 1:** Subdivision according to surgical specialty.

Surgical speciality	Percentage (%)	Number of participants
General surgery	56.4	225
Obstetrics and gynecology	18	72
Plastic surgery	7.7	31
Thoracic surgery	4.7	19
Orthopedics	3.7	15
OHNS otolaryngology-head and neck surgery	2.5	10
Maxillo-facial surgery	1.8	7
Ophthalmology	1.8	7
Urology	1.2	5
Vascular surgery	1	4
Cardio surgery	0.3	1
Neurosurgery	0.3	1
Pediatric surgery	0.3	1
Others	0.3	1
Dentistry	0.0	0
Total		399

At the time of questionnaire administration, 381 residents were equally distributed during the 5 years of surgical training, as showed in Table 2. In 51.9% of cases (*n* = 207), trainees were attending the training program in hospitals in the Northern regions of Italy (Table 3).

**Table 2:** Subdivision of respondents according to year of residency and activities usually performed.

**Attended year of surgical residency (381)**
1st year: 76 (19.95%)
2nd year: 81 (21.26%)
3rd year: 77 (20.2%)
4th year: 75 (19.69%)
5th year: 72 (18.9%)
**Main surgical activity performed (380)**
Elective surgery for oncological pathologies (60.53%)
Elective surgery for benign pathologies (19.74%)
Emergency surgery (13.42%)
Transplant surgery (5.26%)
Surgery which requires post-operative anaesthesiologic care (1.05%)

**Table 3:** Italian regions in which the respondents work (*n* = 399 respondents).

Italian region	Percentage (%)	Number of participants
Lazio	23.55	94
Lombardia	16.54	66
Friuli Venezia Giulia	12.03	48
Emilia Romagna	8.52	34
Veneto	6.77	27
Puglia	6.27	25
Sicilia	5.26	21
Piemonte	5.01	20
Sardegna	3.51	14
Toscana	3.01	12
Calabria	2.76	11
Liguria	2.5	10
Umbria	1.5	6
Abruzzo	1.25	5
Campania	1	4
Valle d’Aosta	0.26	1
Trentino Alto Adige	0.26	1
Basilicata	0.00	0
Marche	0.00	0
Molise	0.00	0

Regarding the type of surgery performed in each center, 230 (60.5%) out of 380 responders worked in surgical oncology units, 75 (19.7%) performed surgery only for benign conditions and 51 (13.4%) worked in an emergency surgery departments. Less than 10% of the participants attended organ transplants departments or worked in a surgical unit requiring post-operative anesthesiologic care (Table 2).

### Impact of COVID-19 second wave on research/didactic and surgical weekly activities

**Figure 1:**
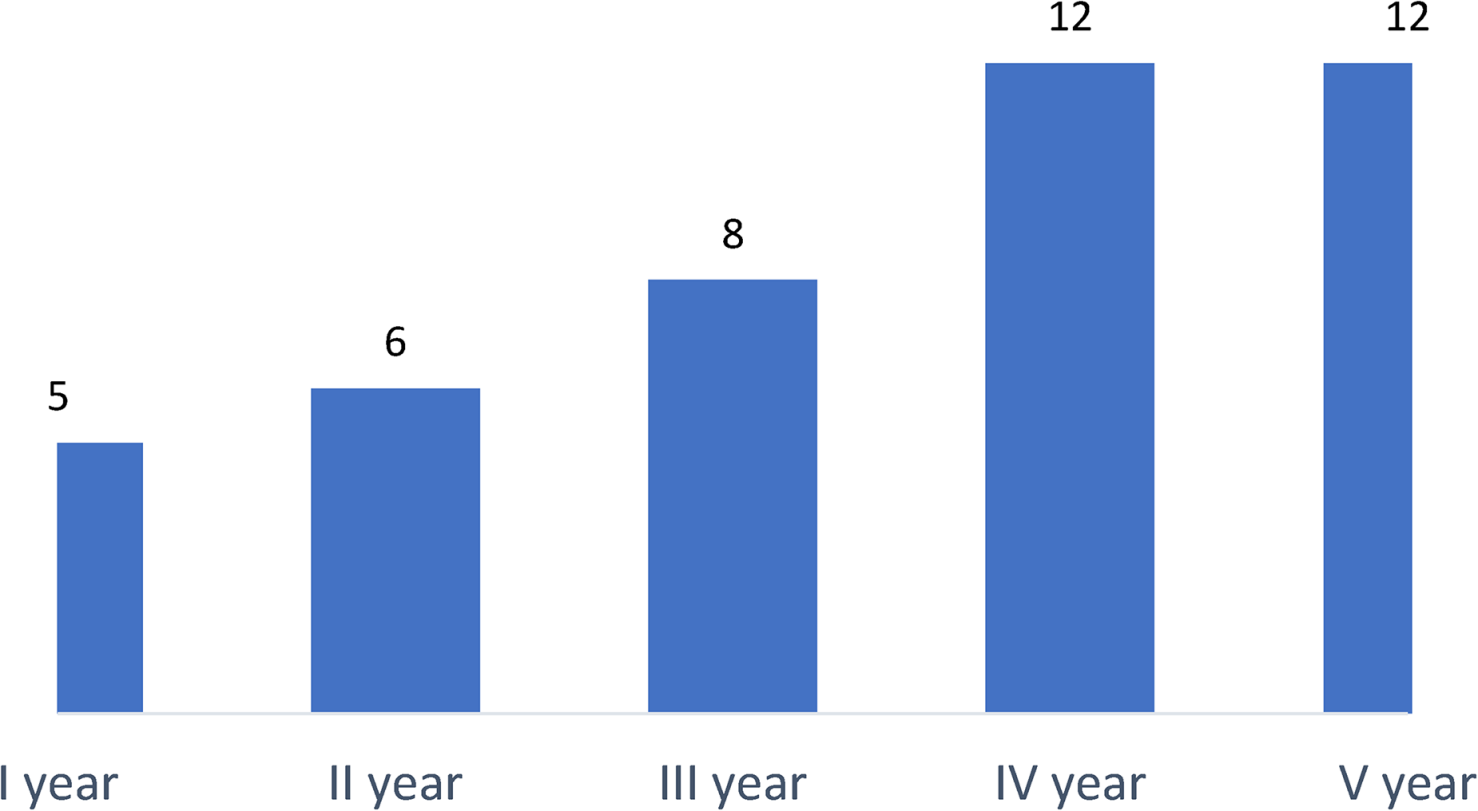
Graphical representation of 43 respondents who left their surgical training divided according to the attended year of residency.

Among 378 responders (94.7%), 335 (88.6%) declared to have continued working in Surgical Units, while a limited proportion of them (5.3%) were moved to a non-surgical unit (Table 4). In this subgroup, 15 (75%) were residents attending general surgery at the fourth year of the training program (35%; Figure 1) and working in northern regions of Italy (90%).

**Table 4:** Impact of pandemic on clinical, surgical and research activities.

Impact of COVID-19 second wave on clinical activities (378)	Impact of COVID-19 second wave on surgical activities (381)	Impact of COVID-19 second wave on training and research activities (380)
Maintenance of usual activity (88.62%)	No relevant modifications (29.66%)	Maintenance of usual activity (36.84%)
Transfer to a non-surgical unit (i.e., COVID ward, internal medicine ward, emergency department) (5.29%)	Reduction of surgical sessions but at least one or more weekly planned surgical sessions (49.61%)	Increase of usual activity (12.37%)
Voluntary Interruption of residency program to take part in COVID-19 emergency unit (in the same hospital or in another) (2.12%)	A significant reduction with less than one surgical session per week (15.49%)	Decrease of usual activity (30.53%)
Interruption of all clinical activities beforehand (0.26%)	Complete interruption of all surgical activities (3.41%)	Interruption of usual activity (4.74%)
Interruption of all clinical activities because of feeling COVID related symptoms (0.79%)	Increase of surgical activity (1.84%)	Usually not performed this activity (15.53%)
Interruption of all clinical activities following the residency program board directives (2.91%)		

Considering the impact of second COVID-19 pandemic wave, there was a statistically significant difference in residents maintaining the usual activity when comparing those belonging to the first 3 years and at last 2 years of the training program (*p* = 0.019) (Table 5).

**Table 5:** Impact of COVID-19 second wave on active participation to clinics according to the attended year of residency.

	I–III (%)	IV–V (%)
I maintained my usual activity	91.8	83.5
I have been moved to a non-surgical unit	3.9	7.5
I voluntarily interrupted my Residency Program to take part of a COVID-19 emergency unit	0.9	4.2
I interrupted all clinical activities	0.4	0.0
I interrupted all clinical activities because I started feeling COVID-19 related symptoms	0.0	2.1
I interrupted all clinical activities following the residency program board directives	3	2.7

Considering the impact of the pandemic on surgical activities, 189 out of 381 responders (49.61%) reported a reduction of surgical sessions despite one or more planned surgical procedures per week; in 29.6% of cases there were no relevant changes in routine surgical activity; a complete interruption of surgical activities occurred in 3.41% of cases; Interestingly, seven residents (1.84%) declared to have performed an increased number of surgical procedures (Table 4).

### Influence of COVID-19 second wave on training and research

**Figure 2:**
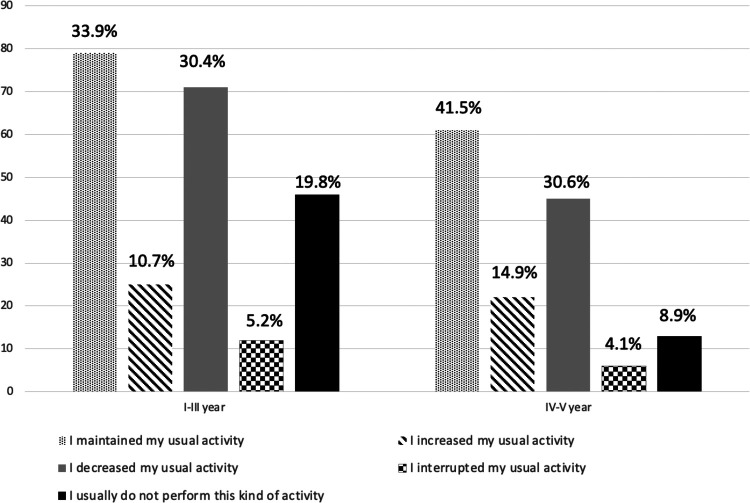
Impact of COVID-19 second wave on training and research activities according to attended year of residency.

Concerning didactic and research activities, among 380 responders (95.2%), 140 (36.8%) declared to have maintained the usual activity, while 116 (30.5%) reported a reduction. Interestingly, 59 residents (15.5%) reported not being involved in didactic and research activities even before COVID-19 pandemic. Forty-seven residents reported an increased activity, 25 of them (53.2%) attending the first 3 years of the residency program (Figure 2). Most of the participants (250, 65.7%) carried out their didactic and research activities in different fields, while 121 (31.9%) and 9 (2.4%) were partially or totally focused on research or didactic activities related to COVID-19 pandemic.

### Influence of COVID-19 second wave on the surgical training program organization

During the period considered in this survey, the surgical training program was not substantially modified for most participants (74.65%); 5.93% of responders (*n* = 22) declared an improvement in training with virtual reality and 8.63% of them in using surgical simulators. Notably, 57 participants (15.36%) reported following “remote” training courses led by experts in a specific field.

Among 371 respondents, 124 (33.42%) reported a complete interruption of surgical training. In 43.13% of cases there was a decrease of the training program activities, although 54 participants (14.56%) defined the training program as “sufficient”. On the other hand, according to 28 respondents (7.55%), the program remained unchanged with respect to before the COVID-19 pandemic; surprisingly, the surgical program was considered improved for 5 residents (1.35%).

**Figure 3:**
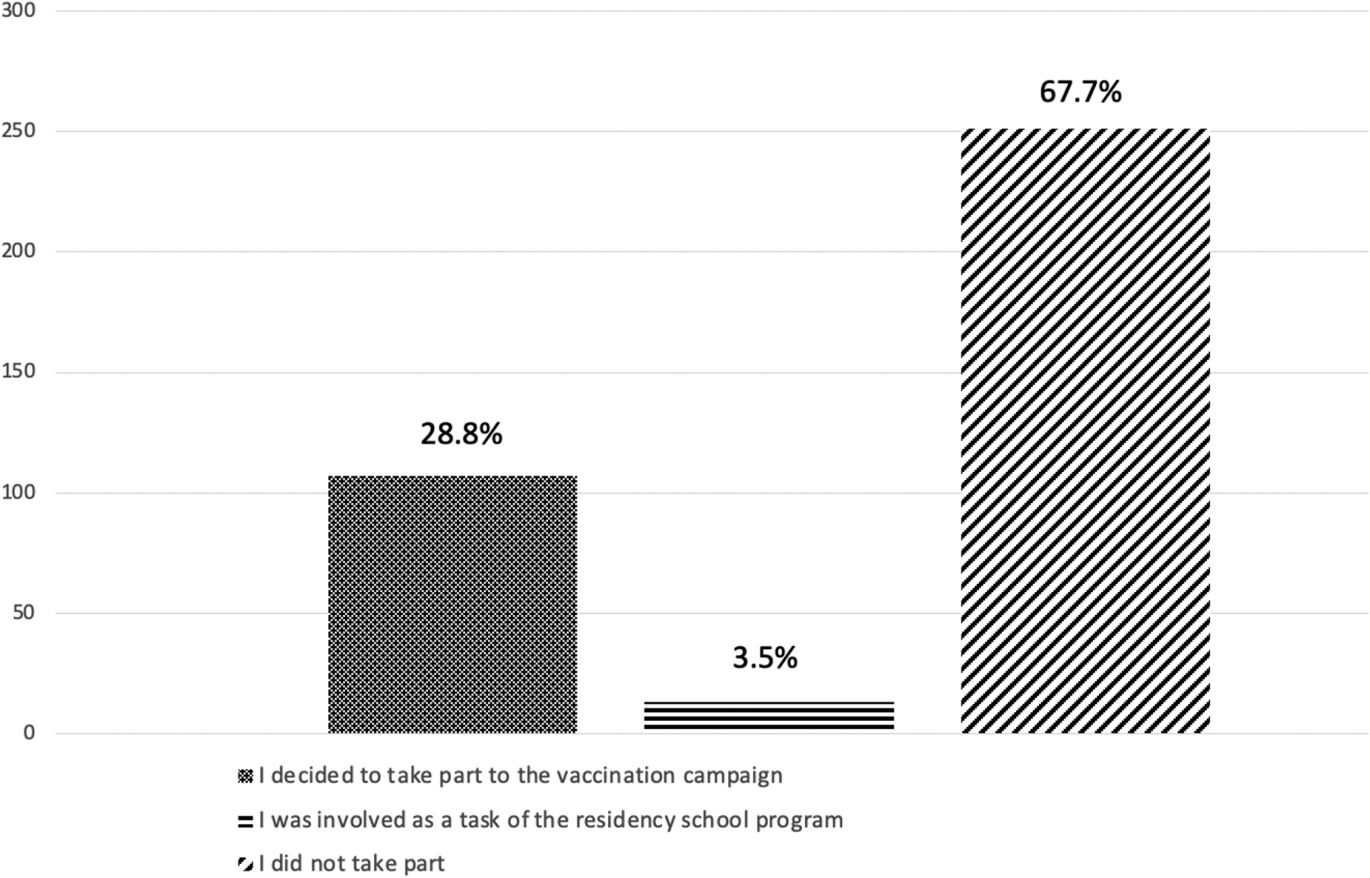
Involvement in national vaccination campaign.

Out of 371 residents, 107 (28.8%) decided to take part in the national COVID-19 vaccination campaign, and 13 (3.5%) participants were involved as part of the residency school program. Nevertheless, most trainees (67.7%) did not take part to the national vaccination campaign (Figure 3).

The residency program resulted improved or partially improved for 6.5% (24 answers) and 40.7% (151 respondents) respectively. However, the program was considered “not improved” by 196 residents (52.8%) completing this survey.

## Discussion

During the first COVID-19 pandemic wave, several articles have analyzed the impact of COVID-19 in different educational teaching programs (18). As reported by Aziz et al., a positive impact of COVID-19 pandemic was related to an improvement in resident educational programs; particularly, these authors reported that residents had a shift to online lessons, leading to global increase in overall teaching time during the pandemic (19).

Unfortunately, less attention has been paid to training programs during the second COVID-19 pandemic wave, as demonstrated by the lack of reports on this topic. This situation can also be highlighted by the number of responses to this survey, which were about half compared to the previous proposal during the first COVID-19 pandemic wave (10).

General surgery residents seem to be sensitive to this topic; this has been underlined by their extensive participation to this survey, making general surgery the most common surgical specialty performed by the participants (Table 1). During the first COVID-19 wave, the problems related to COVID-19 pandemic involved, in particular, responders from the northern regions of Italy (deeply affected by first months of pandemic); notably, also the findings obtained in the current survey confirmed this trend (51.9% of cases); nevertheless, analyzing data from specific regions, 23.3% of the responses were working in the Lazio region (Table 3), one of the most affected areas in Italy.

In comparison to the last survey (10), experience in terms of resource management developed during the first wave of Covid-19 has led to a decreased reallocation of surgical specialists to a medical area from 14.8% to 5.3%. Surprisingly, a higher number of older trainees were reassigned to non-surgical wards compared to younger residents (*p* = 0.019). This finding, in contrast with the previous survey, could be partly explained by to the lower pressure exerted by COVID-19 pandemic on the health care system, which, therefore, led to a reduced workload, thus allowing the recruitment of staff with more experience (20).

Further confirmation of the improved capacity of the health care system to deal with the pandemic is represented by the decreased number of centers in which surgical activity has been suspended and by a relative increase in those able to maintain their usual surgical activity (21). Concerning the participation to surgical activity, trainees reported a general decrease in attendance to surgical procedures, although in very few cases a complete interruption of the operating activity was observed (Table 4).

Unfortunately, research activity did not benefit from the experience of the first COVID-19 pandemic wave. In fact, 30.5% of respondents to our survey reported a decrease in their research activity. Furthermore, a high percentage (about 33%) of residents declared that their scientific activity was mainly focused on COVID-19. These findings seem to show how in the past 2 years, scientific research in the surgical field has been negatively influenced.

A positive aspect recorded during the second COVID-19 wave was represented by the lack of significant impact on the surgical programming, differently from the previous wave.

Overall, the COVID-19 pandemic greatly affected residency educational programs, particularly those related to surgical areas and organized by centers with reduced clinical experience and case volume (22). The experience from the first wave showed the importance of having a flexible surgical training program due to multiple emergent aspects related to pandemic. The possibility to practice on a simulator has been demonstrated to be crucial, especially at the beginning of training (23). From data reported in this survey, unfortunately, 74.6% of respondents reported that training activities during second wave did not have a relevant improvement in comparison to the previous experience during the first COVID-19 pandemic wave. Improvements to training were reported by some participants, as the introduction of virtual training (5.93%), the adoption of surgical simulators (8.63%), and remote training (15.36%).

The growth of several alternative didactic approaches, such as webinars, e-group discussions, educational videos, podcasts, telemedicine, virtual and augmented reality simulation (especially with the presence of a trainer and not self-driven), represented a positive novelty; Interestingly, several authors advise their incorporation into standard surgical training curricula (11, 24–27). These alternative didactic approaches may represent a good tool contributing to overcome the lack of training and hopefully could be introduced in the education program after the pandemic period.

As further evidence of limited learning flexibility of the surgical training system during COVID-19 pandemic, 33.4% of respondents reported a new interruption of surgical activities during the second wave. The low adhesion by some trainees (only 28.8%) to take part in vaccination programs can be considered as a lack of efforts done by the residency programs in underlining the importance of managing the national emergency. It should also be reported, however, that the vaccination campaign, like other initiatives to fight COVID-19, have been interpreted by some organizations as an opportunity to obtain earnings, thus preventing its broad adhesion among healthcare workers. Considering the importance of vaccines for the restoration of elective surgical activity, there should be serious changes in this direction (28).

The present study has some limitations. The heterogeneity of the sample, in terms of experience and demographics, and the impossibility of quantifying the number of trainees who received the survey represent the major limitation. Another possible limitation is that 20% of responders was at the first year of residency with limited knowledge of the level of surgical and research activities before COVID-19. Furthermore, some items did not receive 100% of responders.

## Conclusion

Our study shows how the residency programs were considered improved by about half of the respondents which, undoubtedly, represents an unsatisfactory result, especially after the first wave.

Moreover, most of the respondents did not have the opportunity to participate in alternative training programs, such as virtual reality or tele-mentoring. Consequently, further improvements are needed to guarantee the completeness of surgical training even in extreme emergency conditions such as the COVID-19 pandemic.

## Data Availability

The data analyzed in this study is subject to the following licenses/restrictions: privacy. Requests to access these datasets should be directed to arcangelopicciariello@gmail.com.

## Appendix A

SPIGC Working Group

**Collaborators to be indexed:**

**Executive Committee:** Federico Berton, Luigi Conti, Giampaolo Formisano, Angelo Iossa, Michele Maruccia, Andrea Mazzari, Luigi Oragano, Francesca Ratti, Matteo Serenari, Alberto Settembrini, Pasquale Sirignano, Domenico Soriero, Carlo Vallicelli, Giuseppe Vizzielli.

**Regional Lead:** Ruggero Dimonte (Puglia), Stefano Cianci (Sicilia), Marco Giovenzana (Lombardia), Geraldo Palmieri (Emilia-Romagna), Edoardo Pasqui (Toscana), Marco Petrillo (Sardegna), Luca Portigliotti (Piemonte), Daniele Sambucci (Triveneto), Giuseppe Sena (Calabria), Marco Sparavigna (Liguria).

**Dissemination Committee**: Giordana Bettini, Gianfranco Fanello, Paolo Mendogni, Lorenzo Monteleone, Nicoletta Pia Ardò, Pasquina Tomaiuolo, Giovanni Tomasicchio, Nicola Paradiso, Rigers Dibra, Giuseppe Trigiante, Agnese Dezi, Ludovico Carbone,Sara Negrello, Mattia Di Bartolomeo, Romeo Patini, Alberto Vito Marcuzzo, Alberto Campione, Giovanni Comacchio, Giacomo Murana, Martino Antonio, Mattia Manitto, Giuseppe Galzerano, Carlo Di Marco, Francesco Velluti, Gianmauro Berardi, Andrea Romboli, Federica Perelli, Jacopo Weindelmejer, Domenico Tamburrino, Alessandro Calarco, Luigi Losco, Eleonora Nacchiero, Rossella Elia, Federico Lo Torto, Giovanni Vicenti, Vincenzo Pappalardo, Dafne Pisani, Graziano Palmisano, Debora Brascia, Luigi Troisi, Federica Renzi, Fabio Melandro, Silvia Pecere, Carlo Gazia, Gregorio Di Franco, Gaetano Romano, Alberto Bolletta, Emanuele Botteri, Giovanna Di Meo, Sonia Chiappetta, Ilaria Sgaramella, Francesco Pennestri, Antonella Girardi, Donatella Mariniello, Marco Marcasciano, Michele Telegrafo, Simona Fracomeni, Francesca De Paoli.
